# Is Certolizumab Pegol Safe and Effective in the Treatment of Patients with Moderate to Severe Crohn’s Disease? A Meta-analysis of Controlled Clinical Trials

**DOI:** 10.5812/ircmj.11258

**Published:** 2013-08-05

**Authors:** Shekoufeh Nikfar, Solmaz Ehteshami-Afshar, Mohammad Abdollahi

**Affiliations:** 1 Department of Pharmacoeconomics and Pharmaceutical Administration, Faculty of Pharmacy, Tehran University of Medical Sciences, Tehran, IR Iran; 2 Food and Drug Organization, Ministry of Health and Medical Education, Tehran, IR Iran; 3 Faculty of Medicine, Shahid Beheshti University of Medical Sciences, Tehran, IR Iran; 4 Faculty of Pharmacy, and Pharmaceutical Sciences Research Center, Tehran University of Medical Sciences, Tehran, IR Iran

**Keywords:** Meta-analysis, Certolizumab Pegol, Crohn’s Disease

## Abstract

**Background:**

Tumor necrosis factor-α (TNF-α) antibodies are currently used in patients with moderate to severe Crohn’s disease (CD) who are unresponsive to conventional therapies. Certolizumab pegol (Cp) is one of the anti-TNF-α agents introduced for the management of CD and rheumatoid arthritis.

**Objectives:**

The aim of this meta-analysis is to assess the efficacy of Cp in inducing clinical response and remission in CD and the associated adverse events. The effect of Cp in terms of CD patients’ C-reactive protein (CRP) level was also studied.

**Patients and Methods:**

Literature was searched for studies investigated the efficacy of Cp on inducing clinical response and maintaining remission in the patients with CD between 1966 and July 2012.

**Results:**

Among 165 potentially relevant studies, six with a total of 1695 patients met the inclusion criteria and were meta-analyzed. In comparison to control groups, patients who received Cp had a relative risk (RR) of 1.38 with absolute risk reduction (ARR) = 0.12; 95% CI = 0.03 to 0.21), number needed for treatment (NNT) = 9; P < 0.0001 ) for clinical response and RR of 1.54 (ARR = 0.09; 95% CI = -0.0198 to 0.2), (NNT = 12; P < 0.0001) for maintenance of clinical remission and non-significant RR of 1.24 (P = 0.052) for induction of clinical remission. Baseline CRP did not significantly alter the magnitude or response. Adverse events were not significantly different among patients receiving Cp comparing to placebo.

**Conclusions:**

Cp is effective for inducing clinical response and maintenance of clinical remission in patients with moderate to severe CD with similar side-effect profile as the control arms.

## 1. Background

Crohn’s disease (CD) is a subtype of inflammatory bowel disease (IBD) that can affect any part of the gastrointestinal tract. CD patients experience a relapsing and remitting course. The main cause of CD still remains unknown, however it is believed that the proinflammatory cytokine tumor necrosis factor alpha (TNF-α) plays a key role in the inflammation associated with CD. Abnormal levels of TNF-α has been found in the blood and other biological fluids of CD patients (1). Various medical treatments have been tested in management of CD such as corticosteroids, immunomodulators and biological therapies. Among them, TNF-α antibodies are administered in severe cases unresponsive to preliminary conventional therapies (1, 2). Since 1998, infliximab (IFX) has been used, however two other anti TNF-α agents, adalimumab (Humira; Abbott Laboratories, Abbott Park, IL) and Certolizumab pegol (Cp or CDP870) (Cimzia; UCB Pharma, Brussels, Belgium) have been recently introduced to the market (3, 4). IFX and adalimumab are IgG1 monoclonal antibodies that bind to TNF-α (5). However, Cp is a pegylated humanized Fab′ fragment of an anti-TNF-α monoclonal antibody which unlike other monoclonal antibodies does not have a Fc portion and therefore does not activate complement system, antibody-dependent cellular cytotoxicity, or apoptosis in vitro (4-6). The addition of two molecules of polyethylene glycol to the antibody fragment increases the plasma half-life to approximately 2 weeks, and reduces the required frequency of dosing (7, 8). Cp is also effective for rheumatoid arthritis (9) and it has been approved by US food and drug administration (FDA) (10). The previous meta-analyses of randomized placebo controlled trials which evaluated the efficacy of all anti TNF-α agents consisted information regarding Cp (3, 11) but only three and two trials of Cp was included respectively. In a meta-analysis carried out by Peyrin-Biroulet et al. (3), three trials of Cp were included in subgroup analysis. In a recent meta-analysis of Ford et al. (12), four Cp trials were included however induction of clinical response and analysis based on CRP was not conducted. In another meta-analysis, only the efficacy of Cp in CD patients with 3 included articles was assessed (4). Therefore, we found the need to perform this meta-analysis to provide further results on the efficacy of Cp in inducing clinical response and remission in CD and the associated adverse events. As recently suggested, measurement of baseline C-reactive protein (CRP) besides CD activity index (CDAI) is more helpful in the assessment of patients with CD. Thus, in the present work, we updated our data by including effect of Cp in relation to CRP variations in CD patients.

## 2. Objectives

The aim of this meta-analysis is to assess the efficacy of Cp in inducing clinical response and remission in CD and the associated adverse events. The effect of Cp in terms of CD patients’ C-reactive protein (CRP) level was also studied.

## 3. Patients and Methods

### 3.1. Data Sources

PubMed, Scopus, Web of Science, and Cochrane Central Register of Controlled Trials were searched for studies that investigated the efficacy of Cp in CD. Data were collected from 1966 to July 2012. The search terms were “Crohn’s disease” and “certolizumab pegol” or “CDP870” and “clinical trial” and also their abbreviations were applied. For PubMed, all relevant MeSH terms were used. The final queries were validated by manual review. The reference lists from retrieved articles were also evaluated to make sure all applicable studies were included. The conference proceedings were also searched. The key outcome of interest was clinical response. Induction and maintenance of remission were secondary outcomes of interest.

### 3.2. Study Selection

Studies that investigated the effectiveness of Cp in CD were considered. Three reviewers independently reviewed the title and abstract of each article to eliminate duplicates, reviews, case studies, trials that did not have institutional review board approval and uncontrolled trials and those published in languages other than English. Studies which were clinical trials were included. Disagreements between reviewers were resolved by consensus. Data on patients’ characteristics, therapeutic regimens, dosage, sample size, trial duration, and outcome measures were extracted.

### 3.3. Definition of the Terms

Clinical response in all the articles was defined as a decrease of more than 100 points from the baseline in CDAI and remission as defined a CDAI of ≤150 (7, 9, 10, 12). Improvement in health related quality of life (HRQoL) defined by an increase of at least 16 points in the total score of IBD questionnaire (IBDQ) compared with the score recorded during first week of the studies (7). Maintenance of the improved HRQoL was defined as total IBDQ score ≥ 170 points (8). CRP level is divided to high CRP (≥ 10 mg⁄ L) and low CRP (< 10 mg⁄ L) (9).

### 3.4. Assessment of Trial Quality

Jada score quality assessment method for clinical trials has been applied to evaluate the quality of included studies (13) (Table 1). This method is judging clinical trials based on randomization: in case of randomized it score one point and if the way of randomization is described another point can be added to total score. Other base of evaluation is blinding: for blinding and appropriately description of it for each one point and finally one more score for explanation of withdrawals and dropouts. The quality scale ranges from 0 to 5 points with a low quality report of score 2 or less and a high quality report of score at least 3.

**Table 1.** Jadad Score of Clinical Trials

References	Randomization	Blinding	Withdrawals and dropouts	Total Jadad score
**Schreiber 2005**	1	1	1	3
**Winter 2004**	2	1	1	4
**Sandborn2011**	2	1	0	3
**Sandborn 2007**	2	1	1	4
**Schreiber 2007**	2	1	1	4
**Rutgeerts 2008**	2	1	1	4

### 3.5. Statistical Analysis

Data from selected studies were extracted in the form of 2×2 tables. All included studies were weighted and data of patients who received Cp were pooled. Data were analyzed using Stats Direct (2.7.9). Relative risk (RR) and 95% confidence intervals (95% CI) were calculated using the Mantel-Haenszel and Der Simonian-Laird methods. The Cochran Q test was used to test heterogeneity. The event rate in the experimental (intervention) group against the event rate in the control group was calculated using L'Abbe plot as an aid to explore the heterogeneity of effect estimates. Funnel plot analysis was used as publication bias indicator.

## 4. Results

**Figure 1.**
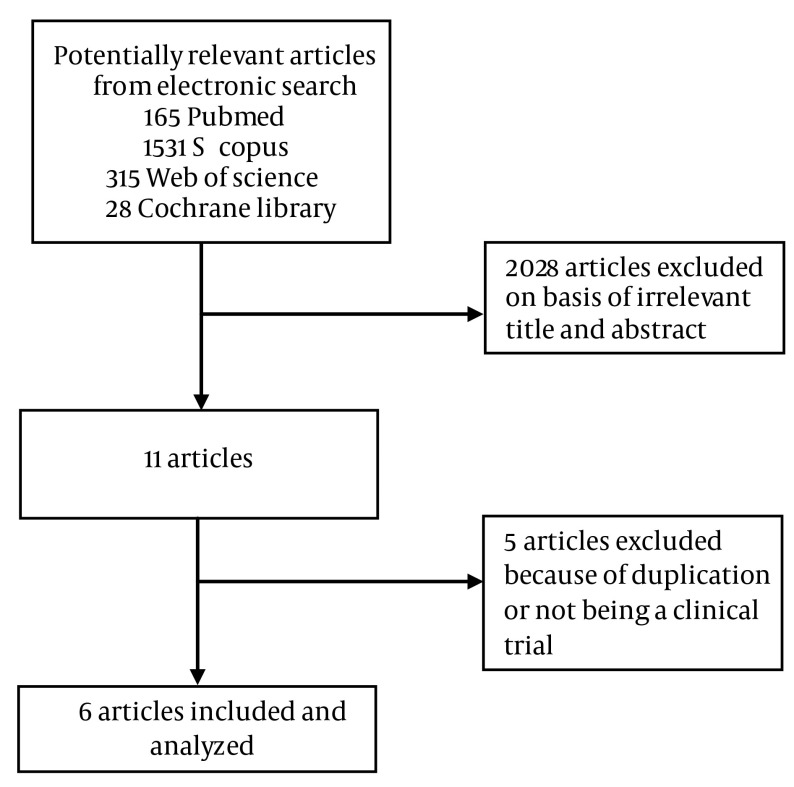
Flow Diagram of the Study Selection Process

The electronic searches yielded 165 potentially relevant studies from PubMed, 28 from Cochrane Central, 315 from Web of Science, and 1531 from Scopus. Of these, 11 articles were inspected in full text. Three reports were excluded because of duplication. Two studies were considered excluded because in Hanauer et al. (14) the impact of prior IFX therapy on the clinical response to Cp was assessed. In Sandborn et al. (15) patients with relapsed CD were included. Five randomized clinical trials (RCTs) were included in the meta-analysis. A total of 1695 patients with CD were randomized to receive Cp or placebo (5-9) (Tables 2, 3, 4 and Figure 1). All the trials were multicenter studies. One of the studies analyzed the same group of patients and thus only that part of the results that assessed the effect of Cp on the remission based on IBDQ and it was not mention in the previous article, was included (16).

**Table 2.** Results of the Studies Included in the Meta-analysis Cp : Certolizumab Pegol

	Clinical response		Remission		Adverse event	
	placebo	Cp 400 mg	placebo	Cp400 mg	placebo	Cp400mg
**Schreiber 2005**	26/73	32/73	17/73	19/73	51/73	48/73
**Winter 2004**	13/24	16/26			15/24	17/26
**Sandborn 2011**	71/209	87/215	53/209	68/215	114/223	100/215
**Sandborn 2007**	115/327	87/325	32/326	47/327	260/329	269/331
**Schreiber 2007**	76/210	135/215	60/210	103/215	143/212	140/216

**Table 3.** Characteristics of included studies in meta-analysis

	Inclusion criteria	Exclusion according to Previous TNF-α usage	Concomitant therapy	Drugs	Article type	Duration	Number of patients	
							placebo	Cp 400 mg
**Schreiber 2005**	moderate to severe CD, CDAI score of 220–450 points	receipt of other anti-TNF-α therapy with a biologic agent within 12 weeks of screening or treated previously with any anti-TNF-α agent and either had experienced an infusion reaction or confirmed to be associated with an immune response, or had showed a lack of clinical response to the first dose.	stable dose of aminosalicylates, antidiarrheal, anti-infectives, metronidazole, ciprofloxacin, codeine and derivatives, immunomodulators azathioprine 6-mercaptopurine , methotrexate Glucocorticoids (overall) ,Systemic glucocorticoids ,budesonide	Cp^a^100 mg, 200 mg, 400 mg, or placebo	phase II, multicenter, randomized, double-blind, placebo-controlled, parallel-group, dose-response study	12 weeks	73	73
**Winter 2004**	moderate to severe CD, CDAI score of 220–450 points	previous treatment or participation in a clinical trial with anti-TNF-α therapy within 12 weeks of screening	azathioprine, methotrexate and mercaptopurine (6-mercaptopurine); antibiotics, sulfasalazine (sulphasalazine), mesalazine, olsalazine, pentasa or similar analogues; corticosteroids and topical anorectal treatments.	Cp (1.25 5, 10 or 20 mg/kg body weight) or placebo	phase II, single-dose, randomized, double-blind, placebo-controlled, parallel-group, multicentre	4 weeks	24	26
**Rutgeerts 2008**	moderate to severe active CD		Concomitant medication was allowed	Cp (100, 200, or 400 mg) or placebo	multicenter, randomized, double-blind, placebo controlled		73	72
**Sandborn 2011**	active CD,CDAI score of 220– 450 points	prior treatment with any anti-TNF-α agent or other biological agent and those receiving intravenous corticosteroids	oral corticosteroids, immunosuppressants, antibiotics, 5-aminosalicylic acid analogues, topical anorectal treatments, antidiarrheal, analgesics, and probiotics.	Cp400 mg or placebo	multicenter, randomized, double-blind, placebo controlled trial	6 weeks	215	223
**Sandborn 2007**	CD for at least 3 months with a CDAI score of 220 to 450 points	received any anti-TNF-α agent within the previous 3 months or who had had a severe hypersensitivity reaction or a lack of response to the first dose of another TNF-α antagonist	stable doses of 5-aminosalicylates, prednisolone or its equivalent (at a dose of 30 mg per day or less), azathioprine, 6-mercaptopurine, methotrexate, or antibiotics.	Cp 400 mg or placebo	multicenter, randomized, double-blind, placebo-controlled trial	26 weeks	328	331
**Schreiber 2007**	3-month history of active CD,CDAI score of 220 to 450 points	received an anti-TNF-α agent or other biologic therapy within 3 months before enrollment, or who had a severe hypersensitivity reaction or no clinical response after initial dosing with an anti-TNF-α	stable doses of 5-aminosalicylates, 30 mg or less of prednisolone per day (or equivalent), azathioprine, 6-mercaptopurine, methotrexate, and antibiotics.	Cp 400 mg or placebo	multicenter, randomized, double-blind, placebo-controlled trial	26 weeks	212	216

^a^Abbreviation: CDAI, crohn’s disease activity index; Cp, certolizumab pegol; CD, crohn’s disease

**Table 4.** Remission and Clinical Response Based on IBDQ of the Included Studies in Meta-analysis

	IBDQ^a^response		IBDQ remission	
	Placebo	Cp	Placebo	Cp 400mg
**Sandborn 2011**			60/209	79/215
**Sandborn 2007**	108/328	140/313		
**Rutgeerts 2008**			Week 6	Week6
**Hanauer 2010**	90/210	129/214	13/73	28/72

^a^Abbreviations: CP, Certolizumab pegol; IBDQ, Inflammatory Bowel Disease Questionnaire

### 4.1. Clinical Response of Cp Comparing to Placebo in CD Patients

**Figure 2.**
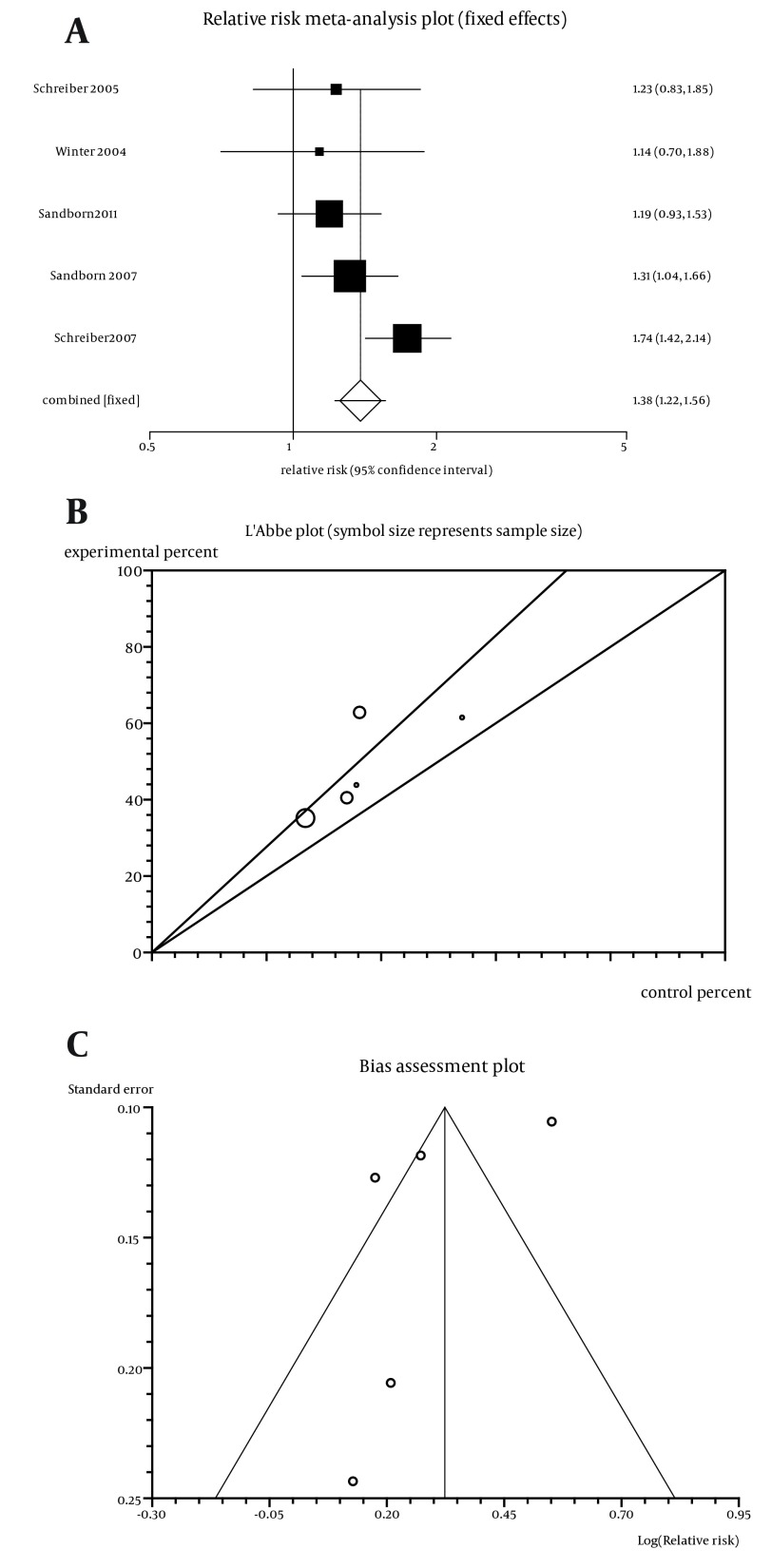
a. Individual and Pooled Relative Risk for the Outcome of “Clinical Response” in the Studies Considering Certolizumab Comparing to Placebo Therapy, b. Heterogeneity Indicators for the Outcome of “Clinical Response” in the Studies Considering Certolizumab Comparing to Placebo Therapy, c. Publication Bias Indicators for the Outcome of “Clinical Response” in the Studies Considering Certolizumab Comparing to Placebo Therapy

The summary RR for clinical response in five trials (5 -9) was 1.38 with a 95% CI of 1.22-1.56 (absolute risk reduction (ARR) of 0.12 (95% CI = 0.03 to 0.21) and number needed for treatment (NNT = 9) and a significant relative risk (RR; P < 0.0001; Figure 2-a). The Cochrane Q test for heterogeneity indicated that the studies are not heterogeneous (P = 0.13, Figure 2-b) and could be combined. Thus the fixed effect model was applied for individual and summary of RR. Regression of normalized effect vs. precision for all included studies for clinical response among Cp vs. placebo therapy was -2.45 (95% CI = -8.61 to 3.72, P = 0.3), and Kendall’s test on standardized effect vs. variance indicated tau = -0.6, P = 0.08 (Figure 2-c). The summary RR for improvement in HRQoL defined by IBDQ in two trials (5, 14) was 1.38 with a 95% CI of 1.21-1.59 and a significant RR (P < 0.0001).

### 4.2.Maintenance of Clinical Remission of Cp Comparing to Placebo in CD Patients

**Figure 3.**
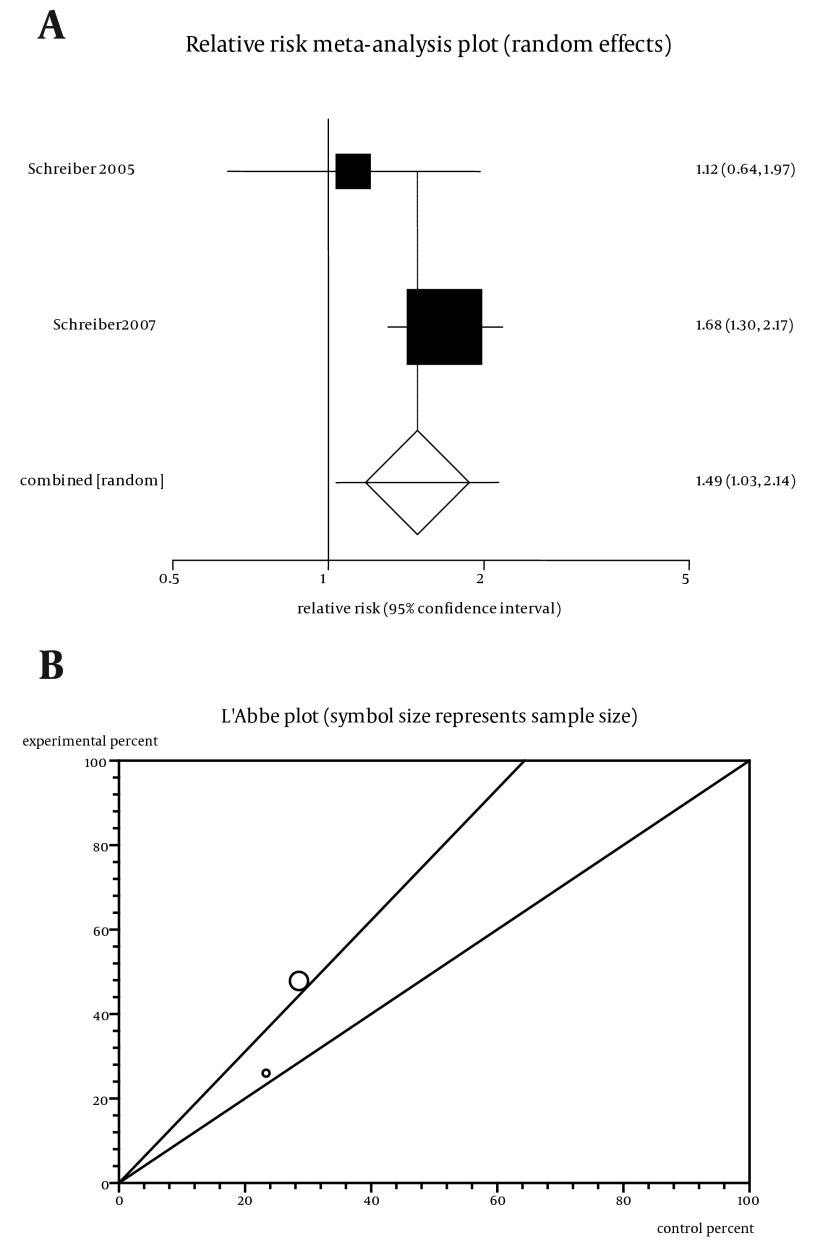
a. Individual and Pooled Relative Risk for the Outcome of “Maintenance of Remission” in the Studies Considering Certolizumab Comparing to Placebo Therapy, b. Heterogeneity Indicators for the Outcome of “Maintenance of Remission” in the Studies Considering Certolizumab Comparing to Placebo Therapy

The summary RR for remission in three trials) (5-7) was 1.54 with a 95% CI of 1.26-1.89, (ARR = 0.09; 95% CI = -0.0198 to 0.2; NNT = 12) and a significant RR (P < 0.0001, Figure 3-a). Heterogeneity has been evaluated by Cochran Q test. The test indicated that the studies are homogenous (P = 0.43, Figure 3-b) but the number of included studies was too few for applying fixed method, thus the random effects for individual and summary of RR was applied. Regression of normalized effect vs. precision for all included studies for maintenance of remission among Cp vs. placebo therapy could not be calculated because of too few strata. The summary RR for maintenance of improved HRQoL defined by IBDQ in two trials (9, 16) was 1.36 with a 95% CI of 1.06-1.73 and a significant RR (P = 0.01).

### 4.3. Induction of Clinical Remission of Cp Comparing to Placebo in CD Patients

**Figure 4.**
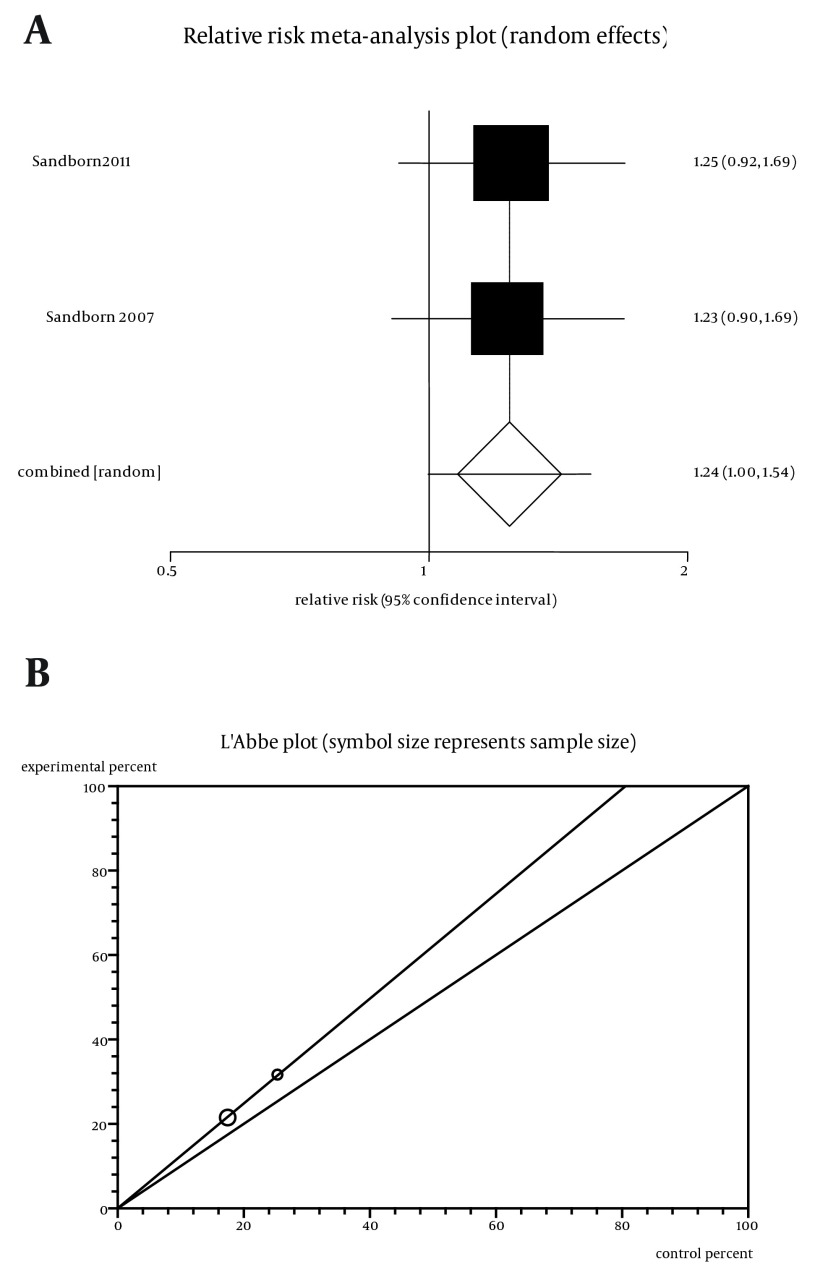
a. Individual and Pooled Relative Risk for the Outcome of “Induction of Remission” in the Studies Considering Certolizumab Comparing to Placebo Therapy, b. Heterogeneity Indicators for the Outcome of “Induction of Remission” in the Studies Considering Certolizumab Comparing to Placebo Therapy

RR for remission in two trials (5, 9) was 1.24 non-significant RR (P = 0.052) with a 95% CI of 0.99-1.54.(Figure 4-a). Test of heterogeneity by Cochrane Q test showed that the trials are homogenous (P = 0.96, Figure 4-b) and could be combined but because of limitation in including studies the random effects was applied. Regression of normalized effect vs. precision for all included studies for induction of remission among Cp vs. placebo therapy could not be assessed because of too few strata.

### 4.4. Any Adverse Events of Cp Comparing to Placebo in CD Patients

**Figure 5.**
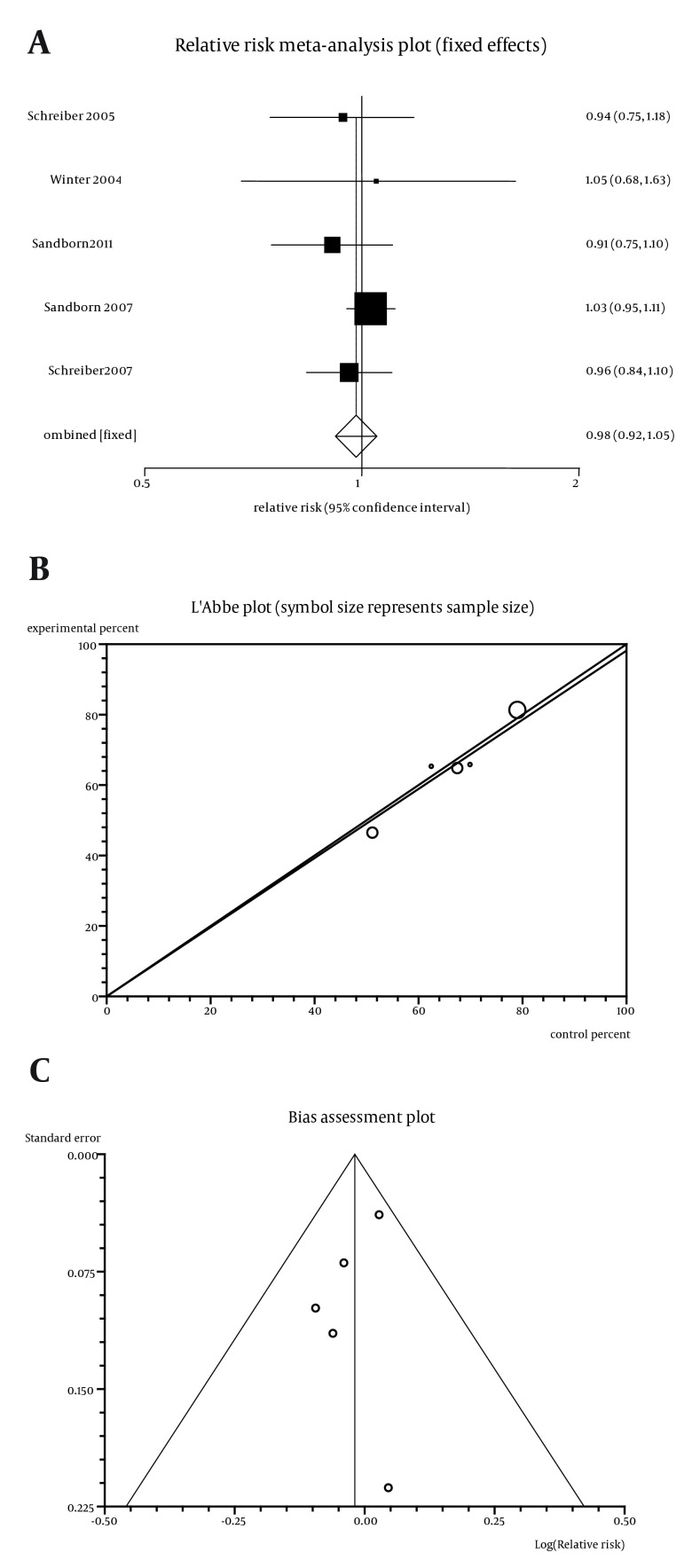
a. Individual and Pooled Relative Risk for the Outcome of “any Adverse Events” in the Studies Considering Certolizumab Comparing to Placebo Therapy, b. Heterogeneity Indicators for the Outcome of “Any Adverse Events” in the Studies Considering Certolizumab Comparing to Placebo Therapy, c. Publication Bias Indicators for the Outcome of “Any Adverse Events” In the Studies Considering Certolizumab Comparing to Placebo Therapy

The summary RR for any adverse events in five trials (5-9) was 0.98 with a 95% CI of 0.92-1.05 and a non-significant RR (P = 0.57, Figure 5-a). The Cochrane Q test for heterogeneity indicated that the studies are not heterogeneous (P = 0.67, Figure 5-b) and could be combined. Thus the fixed effects for individual and summary of RR were applied. Regression of normalized effect vs. precision for all included studies for any adverse events among Cp vs. placebo therapy was -0.78 (95% CI = -2.73 to 1.16, P = 0.29), and Kendall’s test on standardized effect vs. variance indicated tau = 0, P = 0.82 (Figure 5-c).

### 4.5. Clinical Response by Cp in CD Patients Considering High CRP and Low CRP

**Figure 6.**
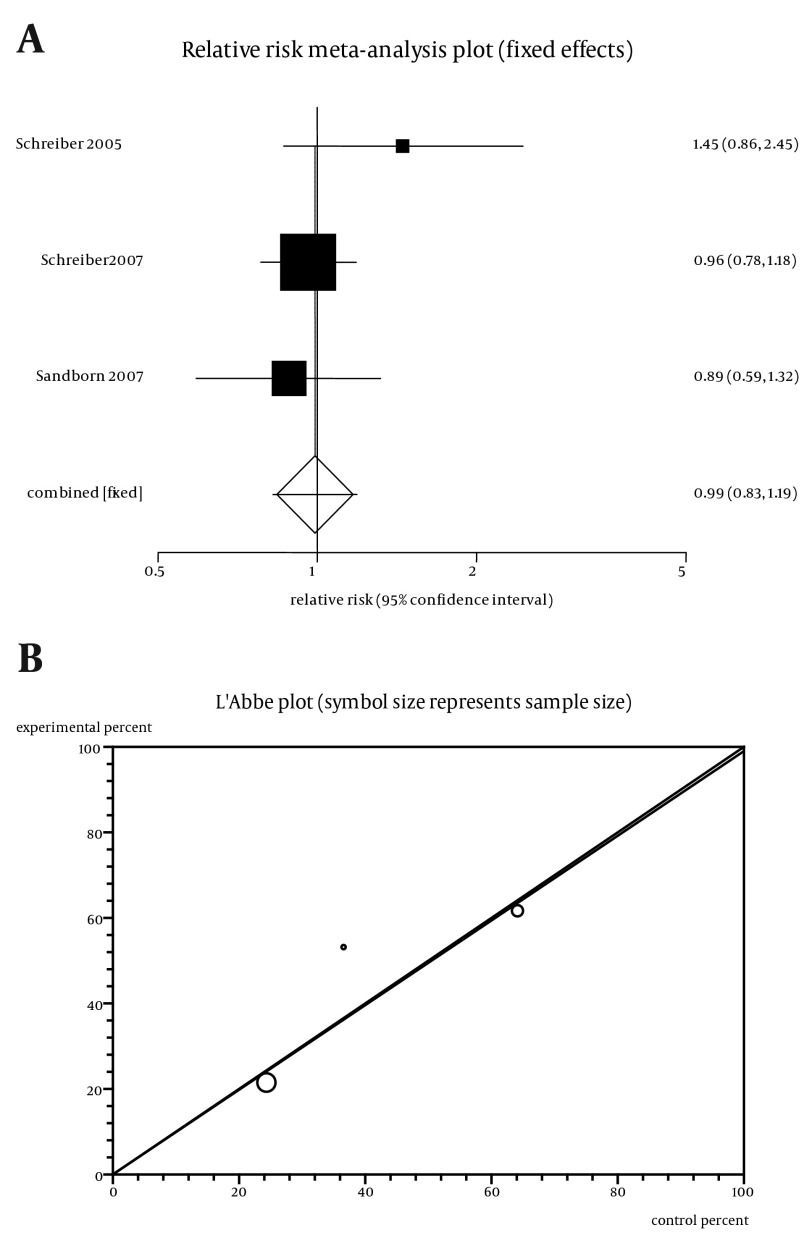
a. Individual and Pooled Relative Risk for the Outcome of “Clinical Response” in the Studies Considering Certolizumab Therapy Comparing Patients with High CRP vs. Low CRP, b. Heterogeneity Indicators for the Outcome of “Clinical Response” in the Studies Considering Certolizumab Therapy Comparing Patients with high CRP vs. low CRP

The summary RR for clinical response in three trials (5-7) was 0.99 with a 95% CI of 0.83-1.19 and a non-significant RR (P = 0.92, Figure 6-a). The Cochrane Q test for heterogeneity indicated that the studies are not heterogeneous (P = 0.29, Figure 6-b) and could be combined. Thus the fixed effects for individual and summary of RR were applied. Regression of normalized effect vs. precision for all included studies for clinical response of Cp therapy among patients with high CRP vs. low CRP could not be calculated because of too few strata.

## 5. Discussion

Although Cp received approval for use in adult CD patients from the US FDA, it has not yet been approved by the European Medicines Agency (EMA) for that indication. This meta-analysis revealed that Cp is significantly more effective than placebo in inducing clinical response and maintaining remission in CD but not in inducing the remission. A total of 1695 patients with moderate to severe active CD, defined by CDAI were included in this meta-analysis. (5-9). There were several limitations for this meta-analysis. Initially, there were differences in study designs such as various duration of treatment with different aims (induction, maintenance), and use of different doses of Cp. However the dosage of 400 mg was chosen for this meta-analysis. For the duration of the study, among different reported results, the primary endpoint was used as reference to extract the outcomes. For assessing the maintenance, responders to the initial induction therapies were included and those with no response in the initial phase were not analyzed in this study. Moreover, although the articles included in this meta-analysis were all RCTs with high quality score and it was assumed that they were all double blinded, because Cp and placebo did not have the same color or viscosity, full blinding was not possible (7). Also, the teams of investigators in the trials are almost the same; however, the centers and years of performing the investigations were different. This issue could raise the bias, because the methods of the studies were all the same and it would cause a decrease in external validity. There were other trials that were not included in this meta-analysis. For instance, the study of Hanauer et al. (14) who evaluated the effect of prior IFX therapy on the response of Cp was not included because the patients were subgroup of Schreiber et al. (6) study that might cause duplication. For the same reason, the trial of Schreiber et al. (17) was excluded. Also, the study of Sandborn et al. (15) was not included because it evaluated the effect of re-induction with Cp in CD patients. Several meta-analyses assessed the efficacy of the anti-TNF-α therapy for inducing and maintaining clinical response and remission in patients with CD. However few meta-analyses were conducted for assessing Cp. Peyrin et al. (3) performed a meta-analysis by comparing the effectiveness of six anti-TNF-α agents (IFX, adalimumab, Cp, etanercept, onercept, and CDP571) with a placebo in CD. Three trials of Cp were included and subgroup analysis showed that Cp is more effective than placebo for induction of clinical remission at week 4 in patients with CD. The results of Behm’s review (11) showed the efficacy of IFX, adalimumab and Cp (separately) in comparison to the placebo for the maintenance of remission in patients with CD. However, only two studies, concerning Cp were included. In the latest meta-analysis which assessed all the biological therapies, four studies of Cp were included and although in the overall analysis, the anti-TNF-α agents (Cp, adalimumab, and IFX combined) were significantly more effective than the placebo for inducing remission and preventing relapse of CD, the conclusion was that Cp is not effective for inducing remission (12). In the previous meta-analysis, only the efficacy of Cp in CD patients was evaluated by including three articles (4). Their results revealed that Cp is more effective than placebo in induction of response and induction and maintenance of remission. However baseline CRP levels are not predictive of rates of response or remission. Serum concentrations of CRP, an acute-phase protein along with interleukin-6, TNF-α and other proinflammatory cytokines that are produced within the intestinal lamina propria is an indicator of inflammation (7). But it must be remembered that CRP reflects systemic immune response and it is not specific for intestinal inflammation. Mean concentration of CRP in patients with IBD is approximately 1.5 mg/L and increased serum level is an indicator of active disease (7). Thus it was suggested that the measurement of baseline CRP added to CDAI in the assessment of patients with CD might help identifying the patients who could get benefit from anti-inflammatory therapy (7). However, this meta-analysis revealed that clinical response in patients receiving Cp or placebo regarding their CRP did not vary significantly. Thus the response rate did not depend on the baseline burden of inflammation as measured by CRP. The suggestion was that there should be a correlation between ileal disease location and a low baseline CRP concentration and patients with the involvement of terminal ileum had lower levels of CRP (9). Clinical remission regarding CRP was not assessed due to the small number of trials. Due to disease-related concerns, especially the necessitate of undertaking surgeries, HRQoL in patients with CD is poor (16). One of the goals in treating CD is improvement of HRQoL. The HRQol in patients with IBD is measured by a specific IBDQ which assesses the four aspects of patient’s life that are involved in the disease: symptoms directly related to the primary bowel symptoms, systemic symptoms, emotional function, and social function (16). This meta-analysis showed that clinical response and remission on the way that is indicated in IBDQ is significantly better in the patients treated with Cp in comparison to placebo. Although more trials are needed for more precise conclusion, this meta-analysis suggests that Cp might improve HRQoL in CD patients. It was believed that all anti-TNF-α agents would increase the development of serious fungal, bacterial, or viral infections that could be attributed to malignancy by suppression of the immune response (5). However as it was revealed in the meta-analysis of Peyrin-Biroulet et al. (3), anti TNF-α therapy did not increase the risk of adverse events. Any adverse events in patients receiving Cp and placebo were almost similar in all the trials. There is no significant difference between rates of any adverse events occurred in the two groups. However a long-time exposure in large cohorts is needed for assessment of adverse events associated with Cp. Also due to the small number of the data the analysis was carried on any adverse events and not on subgroups of adverse events. With respect to previous review studies, our meta-analysis is the most up-to-date on the effect of Cp in CD. By adding three more trials and studying the side effects and evaluating efficacy regarding their CRP levels, current data are more reliable and comprehensive. However further clinical trials are still needed for more accurate results, especially according to the adverse events of Cp. Also trials comparing Cp with other anti-TNF-α agents are essential.
